# *MED12*-Related Disease in a Chinese Girl: Clinical Characteristics and Underlying Mechanism

**DOI:** 10.3389/fgene.2020.00129

**Published:** 2020-02-27

**Authors:** Chao Wang, Longlong Lin, Yan Xue, Yilin Wang, Zhao Liu, Zicheng Ou, Shengnan Wu, Xiaoping Lan, Yuanfeng Zhang, Fang Yuan, Xiaona Luo, Chunmei Wang, Jiaming Xi, Xiaomin Sun, Yucai Chen

**Affiliations:** ^1^ Department of Neurology, Shanghai Children’s Hospital, Shanghai Jiao Tong University, Shanghai, China; ^2^ Institute of Medical Science, Shanghai Jiao Tong University School of Medicine, Shanghai, China; ^3^ Division of Pediatric Neurology, Department of Pediatrics, University of Illinois and Children's Hospital of Illinois, Peoria, IL, United States; ^4^ Department of Pediatrics, JianNing General Hospital, Fujian, China

**Keywords:** *MED12*, mutation, X-chromosome inactivation, SHH signaling pathway, craniofacial morphology, intellectual disability

## Abstract

The RNA polymerase II transcription subunit 12 homolog (*MED12*) is a member of the mediator complex, which plays a critical role in RNA transcription. Mutations in *MED12* cause X-linked intellectual disability and other anomalies collectively grouped as *MED12*-related disorders. While *MED12* mutations have been most commonly reported in male patients, we present the case of a 1-year-old girl with clinical characteristics similar to *MED12-*related disorders. To explore the clinical characteristics of the condition and its possible pathogenesis, we analyzed the patient’s clinical data; genetic testing by whole-exome sequencing revealed a *de novo* heterozygous mutation (c.1249-1G > C) in *MED12*. Further cDNA experiments revealed that the patient had an abnormal splicing at the skipping of exon9, which may have produced a truncated protein. qPCR showed decreased *MED12* gene expression level in the patient, and an X-chromosome inactivation test confirmed a skewed inactivation of the X-chromosome. The lymphoblast transcription levels of the genes involved in the Gli3-dependent sonic hedgehog (SHH) signaling pathway, namely, *CREB5, BMP4*, and *NEUROG2*, were found to be significantly elevated compared with those of her parents and sex- and age-matched controls. Our results support the view that *MED12* mutations may dysregulate the SHH signaling pathway, which may have accounted for the aberrant craniofacial morphology of our patient.

## Introduction

The RNA polymerase II transcription subunit 12 homolog (*MED12*) gene-expressed protein is involved in the formation of a mediator complex (Allen and Taatjes, 2015) that mediates the transcription of RNA polymerase II and transmits information between RNA polymerase II and the transcription factors (Conaway et al., 2005). Mutations of the *MED12* gene can cause *MED12*-related syndrome, a disease that varies in its clinical manifestations (Charzewska et al., 2018) and currently has at least four different subtypes: Opitz-Kaveggia syndrome (FGS1); Lujan syndrome (LS); Ohdo syndrome, the Maat-Kievit-Brunner type (OSMKB); and nonspecific intellectual disability (NSID) (Graham and Schwartz, 2013; Tzschach et al., 2015). As the *MED12* gene is located on the X-chromosome, related syndromes exhibit X-linked inheritance and—despite the condition’s low incidence—are thus more common among men (Lesca et al., 2013). To date, the literature features only six reported variants in females with a history of *MED12*-related syndrome. While no cases of *MED12*-related syndrome have been reported in China, we recently encountered the case of a 1-year-old girl whose clinical characteristics (delayed development, facial features) were consistent with those of *MED12*-related syndromes. Whole-exome sequencing revealed that the patient had a novel *MED12* mutation. Herein, we present the clinical characteristics of this patient and explore the possible underlying pathogenesis of the disease.

## Materials and Methods

### Compliance With Ethical Standards

This study was reviewed and approved by the Ethics Committee of Shanghai Children’s Hospital. Informed consents were obtained from the parents of the patient and the legal representatives of a sex- and age-matched control group for using their blood samples for genetic analysis. Written informed consents to participate in this study and for publication were provided by the legal representatives of the participants. The study complied with Chinese bioethics laws as well as the Helsinki declaration and its later amendments.

### Exome Sequencing

The genomic DNA of the patient’s nuclear family was sent for exome sequencing. The experimental protocols used were similar to those described in a previous study (Zhang et al., 2018). In short, genomic DNA was isolated from the patient and her parents’ blood and was sheared using the Covaris Ultra Sonicator. Exome capturing was carried out using IDTxGen Exome Research Panel (IDT, USA), and paired-end sequencing was performed on Hiseq 2500 platform (Illumina, Inc., CA, USA) to obtain 150-bp reads. Burrows-Wheeler alignment tool (BWA, version 0.7.15) software was used to align paired-end reads to the NCBI human reference genome (GRCh37/hg19). Samtools (http://samtools.sourceforge.net/) and Pindel analysis software (http://gmt.genome.wustl.edu/packages/pindel/) were used to analyze single-nucleotide polymorphisms (SNPs) and indels relative to the reference sequence. The identified variant was further annotated and filtered by the Ingenuity Variant Analysis (https://variants.ingenuity.com). Common variants were filtered based on their frequencies [minor allele frequency (MAF) < 0.05]. Nonsynonymous changes were then evaluated using the SIFT software (http://sift.jcvi.org) and Polyphen software (http://genetics.bwh.harvard.edu/pph2).

### Agarose Gel Electrophoresis for DNA Sequencing

After electrophoresis, the region of the gel containing the desired size range of DNA was excised, and the DNA was subsequently extracted from the gel and purified for further sequencing. Gel purified products were sequenced with an ABI 3730XL DNA Sequencer (Thermo Fisher Scientific, Inc., MA, USA). The results of sequences were analyzed with DNASTAR software (http://www.dnastar.com/).

### Real-Time Quantitative PCR (qPCR) Analysis for Transcription Levels of *MED12* Gene and Sonic Hedgehog (SHH)‐Signaling Genes

Total RNA from the patient and her parents was extracted using a QIAGEN RNA Preparation Kit (QIAGEN Inc., CO, Germany). cDNA from these samples was subjected to reverse transcription and synthesized using a PrimeScript^™^ Strand cDNA Synthesis Kit/RT Master Mix (Takara Shuzo Co., Ltd.). Primer (A) was designed for the *MED12* gene, and primers (B)–(D) were designed for the three SHH signaling genes: *CREB5, BMP4,* and *NEUROG2*. The qPCR primers used in this study are described in **Supplementary Table 1**. Real‐time qPCR was conducted using a SYBR Premix Ex Taq II (Tli RNase H plus) (Takara Shuzo Co., Ltd) in a LightCycler^®^ 96 Instrument (Roche lnc., AG Schweiz) according to the manufacturer protocols. The relative expression of *MED12* and the transcription levels of the SHH signaling genes (*BMP4, CREB5, and NEUROG2*) in the patient’s nuclear family were investigated with RT-qPCR, as well as in a sex- and age-matched control group. Housekeeping gene *B2M* was used as a relative quantity. Each dataset for the transcription levels was generated from triplicate studies, and the data were presented as mean ± SEM. Statistical analyses of qPCR data were performed for comparison of the means of samples using the 2^-ΔΔCt^ method. Asterisks denote statistically significant differences (Student t test, **P < 0.05, ***P < 0.01).

### X-Chromosome Inactivation Pattern Analysis Based on Human Androgen Receptor Gene Polymorphism

To test the X-chromosome inactivation pattern of the patient and her nuclear family, we carried out a methylation-based analysis on the human androgen receptor (*AR*) gene (Allen et al., 1992), according to a reported protocol (Kassim et al., 2004). Briefly, 200 ng of DNA from peripheral blood cells was digested with HpaII (New England Biolabs, Ipswich, MA, USA) at 37°C overnight followed by enzymatic inactivation by heating at 95°C for 10 min. To control the quality of the test results, we used digested and nondigested DNA samples as a pattern to amplify the *AR*. Separated PCR amplification was carried out on the digested and undigested DNA by using primers specific for the methylation regions of the STR (Short Tandem Repeats) of exon1 in *AR*. The PCR conditions were as follows: 95°C for 5 min, 28× (95°C for 30 s, 62°C for 30 s, 72°C for 30 min, and 72°C for 7 min). Sequences of the oligonucleotides used to amplify the CAG STR of exon1 in the *AR* gene were as follows: forward 5′-GCTGTGAAGGTTGCTTCCTCAT-3′ and reverse 5′-CGTCCAAGACCTACCGAGGAGCTT-3′. The fluorescence-labeled PCR products were denatured at 95°C for 5 min and further analyzed by capillary gel electrophoresis using Image Lab software. Analysis was performed with Sub-Cell GT Agarose Gel Electrophoresis Systems (Bio-Rad Laboratories, Hercules, CA, USA).

## Results

### Clinical Description

**Figure 1:**
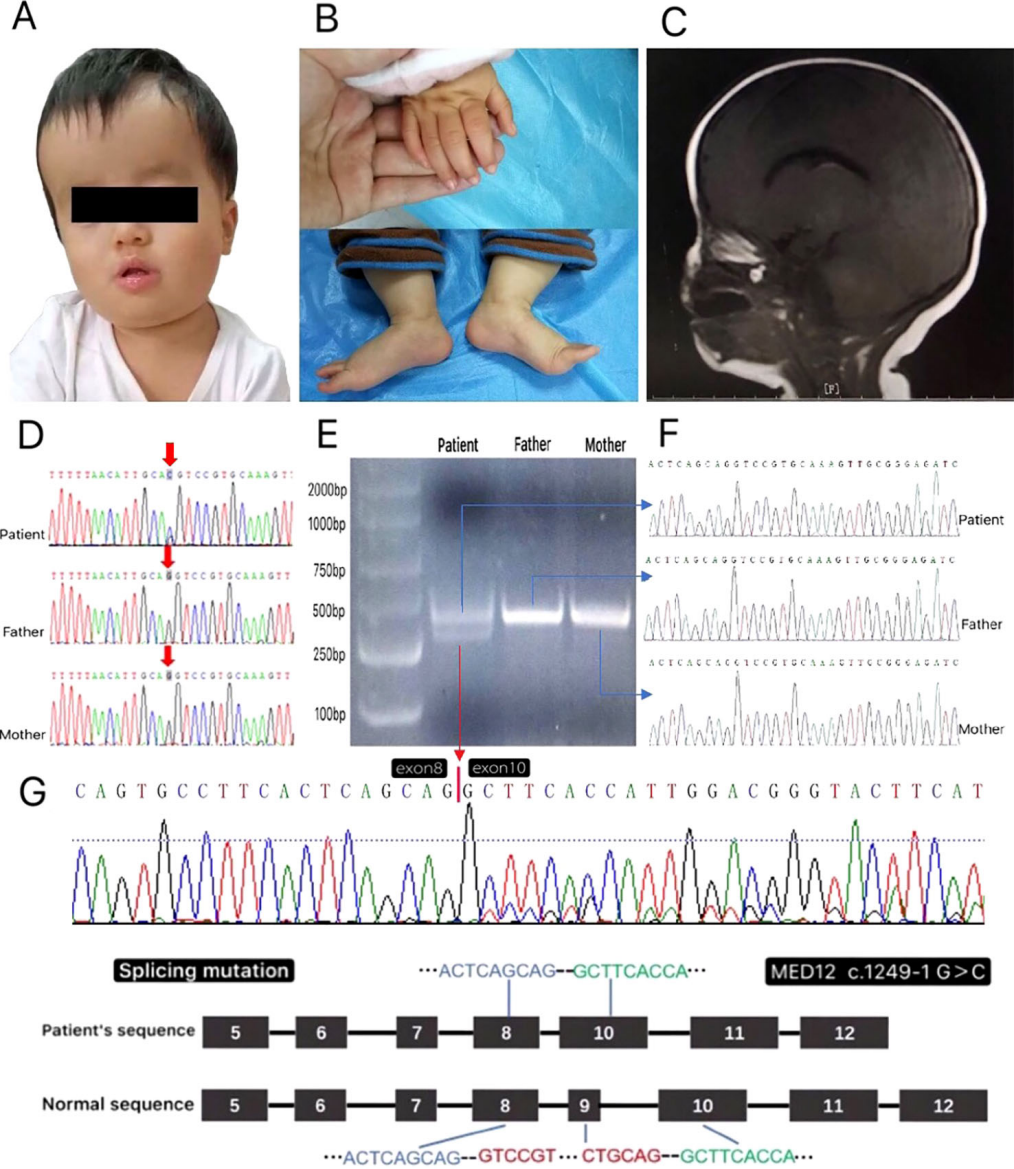
**(A**–**C)** The clinical characteristics of the patient at age 1 were consistent with the phenotype of *MED12*-related disease: **(A)** long forehead, low ear position, prominent nasal bridge, short philtrum, and repaired cleft lip and palate. **(B)** The hypotonia and the overextension joints of the thumb and toe. **(C)** Magnetic resonance imaging (T1- and T2-weighted and T2-fluid attenuated inversion recovery) showed that the corpus callosum was thin. **(D)** The sequences of genomic DNA in the patient’s nuclear family with the detection of a novel heterozygous splicing variant of *MED12* c.1249-1G > C in a girl patient. **(E)** PCR results of cDNA from the patient’s nuclear family. The PCR products of the patient appeared to be two bands: one band like her parents’ and the other truncated band. **(F)** The result of the sequences from the gel purified products of the parents and the patient’s normal band. **(G)** The result of the sequence from the gel purified product of the patient’s truncated band showed the patient had an exon9 skip when compared with the normal sequence of *MED12* gene.

A 1-year-old female patient was brought to the Department of Neurology, Shanghai Children’s Hospital, with the chief complaint of a development delay. She was delivered at term with a birth weight of 3,000 g. There was no history of suffocation at birth or known problems during pregnancy. The parents were not close relatives and had no family history of genetic disease. There was no history of seizures, but the patient had delayed developmental milestones. She could not sit well or speak simple words such as “papa.” The occipito-frontal circumference was 47 cm (1 SD+). The area of the anterior fontanel was 2 cm × 2 cm. The patient had distinctive facial characteristics: a long forehead, low ear position, prominent nasal bridge, short philtrum, and repaired cleft lip and palate (**Figure 1A**). She could raise her head for a long time; however, she could not sit well or stand up by herself. Early psychomotor development was delayed compared with the control group. The girl had generalized hypotonia with overextended toe joints (**Figure 1B**). Magnetic resonance imaging (MRI) showed a thin corpus callosum (**Figure 1C**); there were no other abnormalities. Cardiac ultrasonography showed atrioventricular canal defect, pulmonary stenosis, small internal diameter of the aortic isthmus, and decreased aortic flow velocity. Other laboratory investigations, such as liver and kidney function tests, blood sugar, complete blood count, serum amino acids, lactic acid, pyruvate, creatine kinase, and urine amino and organic acids were all within the normal limits. No abnormalities were found on electroencephalography examination.

### Whole-Exome and Sanger Sequencing

The *MED12* gene (OMIM: 300188; NM_005120.3) c. 1249-1G > C novel heterozygous mutation was detected in the whole-blood genomic DNA of the patient, which was confirmed by Sanger sequencing. The results showed that the parents have no variation at this site (**Figure 1D**). The mutation was present in the intron at a critical splicing position. The mutation seen was rare and not reported in the ExAC, ESP, or 1000G databases (MAF = 0). According to the American College of Medical Genetics and Genomics (ACMG) standards for interpretation of sequence variation, the identified novel *MED12* mutation was categorized as pathogenic and might result in the skipping of exon9.

### Skipping of Exon9 in the cDNA of the *MED12* Gene in the Patient

To test whether *MED12* c.1249-1G > C heterozygous mutation results in exon9 skipping, we prepared cDNA samples from the total RNA extracted from this nuclear family. A pair of primers (**Supplementary Table 1**) located upstream and downstream of exon9 of the *MED12* gene was synthesized. After the PCR products were run, the resulting electropherogram revealed that the patient had two bands, one band like her parents’ and the other truncated band (**Figure 1E**). The PCR product strips of the patient and her parents were purified by gelation and further sequenced. Analysis of the sequence from the gel purified product of the patient’s truncated band showed an exon9 skip when compared with the normal sequence of *MED12* gene (**Figure 1F, G**). The skipping of exon9 in the *MED12* gene was speculated to result in a truncated protein without function.

### The Transcription Levels of *MED12* and SHH‐Signaling Genes in the Patient

**Figure 2:**
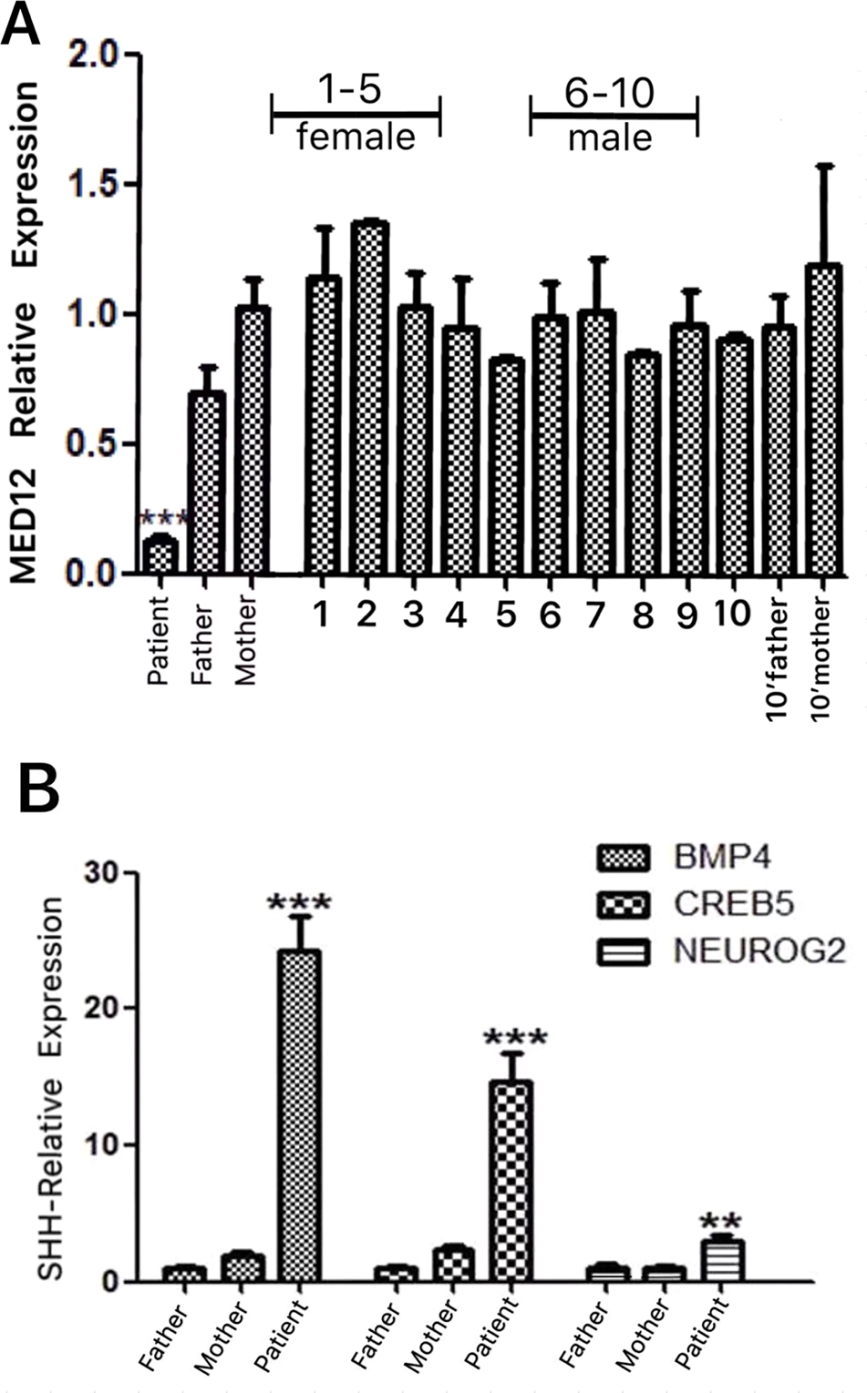
**(A)** The relative expression of *MED12* was investigated with RT-qPCR in the patient’s nuclear family, as well as in a sex- and age-matched control group. The patient exhibited significantly lower expression of the *MED12* gene c.1249-1G > C relative to that of her parents. The discrepancy in the levels of *MED12* was not related to age or sex according to the results of the control group. **(B)** The transcription levels of the sonic hedgehog (SHH)-signaling genes (*BMP4*, *CREB5*, and *NEUROG2*) were investigated with RT-qPCR in the patient’s nuclear family, as well as in a sex- and age-matched control group. The expressions of *BMP4*, *CREB5*, and *NEUROG2* were all significantly enhanced in our patient relative to that in her parents and a sex- and age-matched control group (**Supplementary Figure 1**), suggesting the hyperacitvated output of GLI3-dependent SHH signaling. Each dataset for the transcription levels in **(A)** and **(B)** was generated from triplicate studies presented as mean ± SEM. Statistical analyses of the qPCR data were performed to compare the means of the samples according to the 2^-ΔΔCt^method. Asterisks denote statistically significant differences (Student t test, **P < 0.05, ***P < 0.01).

The results showed that the patient had a significantly lower level of *MED12* expression than did her parents compared with a sex- and age-matched control group (**Figure 2A**). Furthermore, RT-qPCR was used to ascertain the expression levels of *CREB5, BMP4,* and *NEUROG2*. We found that the patient’s transcription levels of these SHH signaling genes were significantly higher than those of her parents and sex- and age-matched controls (**Figure 2B**; **Supplementary Figure 1**).

### Study Skewed X-Chromosome Inactivation Pattern in the Patient

**Figure 3:**
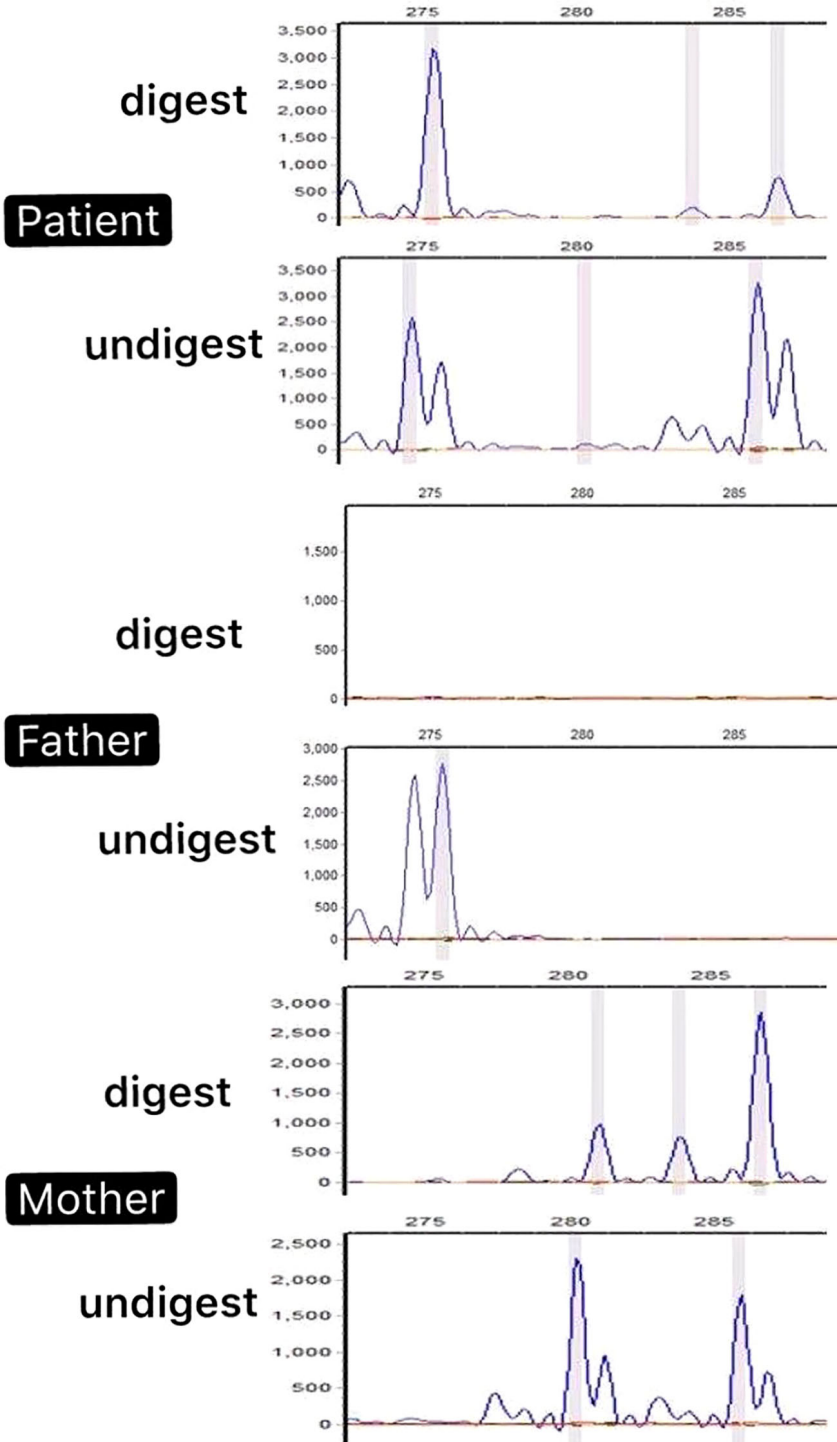
PCR products of the androgen receptor (*AR*) with and without HpaII digestion of the patient’s nuclear family. The peak represents the amplified *AR* allele. The PCR products of the patient derived from the undigested DNA yielded two peaks because of the different numbers of CAG repeats in the two alleles (275 bp from the father and 285 bp from the mother). However, after HpaII was digested, one peak (275 bp) appeared hypermethylated. The results revealed a skewed X-inactivation pattern in the patient with the paternal allele highly inactivated.

Microsatellite PCR products of the *AR* with and without HpaII digestion have shown that the number of repeats within the *AR* gene determines the size of the allele. In this patient, PCR products derived from the undigested DNA yielded two peaks because of the different numbers of CAG repeats of the two alleles, 275 bp from the father and 285 bp from the mother (**Figure 3**). The X-inactivation pattern was considered skewed if the proportion of the two alleles after digestion was at least 20:80 (Giorgio et al., 2017). The experiment revealed that the patient had a skewed X-inactivation pattern (18%:82%) with the paternal allele highly inactivated.

## Discussion

Located on the X-chromosome, the human *MED12* gene consists of 45 GC-repeat-rich exons with a span of 25 kb. The product of the *MED12* gene has been implicated in the formation of a macromolecular complex known as Mediator (Kornberg, 2005), which primarily assists in the transcription of RNA polymerase II and transmitting information between RNA polymerase II and transcription factors (Conaway et al., 2005). Essential for the maintenance of CDK8 molecular activity, *MED12* is considered an important component of the complex (Yin and Wang, 2014). These mediator complexes play important regulatory roles in receptor tyrosine kinases, nuclear receptors, the SHH signaling pathway, and the Wnt signaling pathways (Rocha et al., 2010), as well as involved gene expressions in cell growth, homeostasis, development, and differentiation (Donnio et al., 2017). Mutations in *MED12* can cause diseases with different clinical subtypes.

Mutations in *MED12* at Xq13 causing X-linked intellectual disability (XLID) called *MED12*-related disorders, which include four phenotypes: FGS1, LS, OSMKB, and NSID (Graham and Schwartz, 2013; Tzschach et al., 2015). FGS1 and LS are two allelic XLID syndromes, with mutations in the *MED12* gene, that share some overlapping clinical features, such as dysgenesis of the corpus callosum, macrocephaly, hypotonia, intellectual disability, and behavioral disturbances (Risheg et al., 2007; Schwartz et al., 2007). FGS1 is further characterized by absolute or relative macrocephaly, long forehead, downslanting palpebral fissures, small and simple ears, constipation and/or anal anomalies, broad thumbs and halluces, and characteristic behavior (Opitz and Kaveggia, 1974; Clark et al., 2009). LS can be distinguished by tall stature, long and thin face, hypernasal voice, hyperextensible digits, high nasal root, and short philtrum (Lujan et al., 1984; Schwartz et al., 2007). OSMKB differs from other phenotypes by the facial features such as blepharophimosis, facial coarsening, thick alae nasi, and triangular face. But common spectrums such as skeletal, gastrointestinal, and genital urinary anomalies in the FGS1 and LS are relatively mild or absent in patients with OSMKB (Verloes et al., 2006; Langley et al., 2015). The clinical phenotypes of NSID include intellectual disability and behavioral disorder without significant dysmorphic features or congenital malformations characteristic of FGS1 and LS (Bouazzi et al., 2015; Prontera et al., 2016).

Our patient presented with the clinical features that resembled those associated with both FGS1 and LS: macrocephaly, dysgenesis of the corpus callosum, hypotonia, development delay, long and thin face, hypernasal voice, high nasal root, repaired cleft lip and palate, short philtrum, and hyperextensible digits. Thus, it is more appropriate to consider a “*MED12*-related disorder” than to attribute a definite syndrome.

To date, at least 35 *MED12* genetic variants have been reported relevant to *MED12*-related disorders. All were missense mutations, except one frameshift mutation (Murakami et al., 2019). These previously reported variants were concerned evolutionary conserved sites (**Supplementary Table 2**; **Supplementary Figure 2**).

*MED12*-related disorders are defined as X-linked recessive genetic diseases. In the families of patients with *MED12*-related diseases, carrier females are usually unaffected (Graham and Schwartz, 2013). Up to date, two female carriers with the clinical symptoms (Gurrieri and Neri, 1991; Rump et al., 2011) and six *MED12* variants, c.5898dupC, c.5922G > T, c.2312T > C, c.2545T > C, c.3443G > A, c.514G > C (Lesca et al., 2013; Bouazzi et al., 2015; Prontera et al., 2016; Popp et al., 2017; Charzewska et al., 2018; Murakami et al., 2019), have been reported with intellectual deficiency (ID) and clinical variability. The first time that intellectual disability and aberrant facial development in a female carrier, similar to that of her brother, was reported in 1991 (Gurrieri and Neri, 1991). Later, a single carrier female was reported to have mild learning problems with no cognitive evaluation provided (Rump et al., 2011). A novel frameshift mutation c.5898dupC was then reported in a five-generation family including 10 males and 1 female affected with profound non-syndromic XLID. Variable cognitive impairment was similarly observed in seven heterozygous females in this family. There was no correlation between cognitive function and X-chromosome inactivation profiles in blood cells (Lesca et al., 2013). A novel variant c.5922G > C was reported in three brothers with severe non-syndromic ID, mild dysmorphic features, along with their heterozygous mother with a mild phenotype characterized by borderline ID and language delay (Bouazzi et al., 2015). Another novel missense mutation c.2312T > C was detected in two males and one female in a family, with severe and mild ID, respectively, with phenotype that overlap in part with those of the patients reported on c.5898dupC and c.5922G > C. In this family, the affected female had a completely skewed XCI pattern (Prontera et al., 2016). The three mutations (c.5898dupC, c.5922G > T, and c.2312T > C) with non-syndromic XLID shared the similar condition that ID, clinical manifestation were milder in heterozygous females in the family, but were particularly severe in the affected males (severe ID and absent or deficient language). Another *de novo* missense variant c.2545T > C was reported in a girl with severe but non-syndromic ID. This variant might be pathogenic in female, but remains unclear (Popp et al., 2017). Two mutations, c.3443G > A and c.514G > C, have been described to cause OSMKB. c.3443G > A, a known variant, reported causing OSMKB in males, was first reported in affected females (two affected daughters and their carrier mother) by Charzewska in 2018. XCI analysis in this family revealed a mildly skewed pattern in the mother (82:18) and a skewed pattern in her two affected daughters (100:0 and 85:15) (Charzewska et al., 2018). A novel *de novo* variant c.514G > C causing severe ID and facial dysmorphism in a female patient was also reported consistent with OSMKB. Analysis of XCI revealed a skewed pattern (87:13) (Murakami et al., 2019).

To date, only six MED12 mutations were reported in females with mental retardation and variable dysmorphic features. XCI patterns have not been correlated to phenotype in female carriers and patients for *MED12*-related disorders (Lesca et al., 2013; Bouazzi et al., 2015; Prontera et al., 2016; Charzewska et al., 2018). With the increase of family or sporadic affected females reported in variable clinical expression, the clinical spectrum of *MED12*-related disorder is wider than previously reported. But the mechanism of pathogenesis in females has not been fully elucidated.

Here, and to the best of our knowledge, we present a novel splicing *MED12* gene (OMIM: 300188) mutation c.1249-1G > C that occurred in a female patient with significant clinical symptoms. The cDNA sequencing exhibited an exon9 skip in the patient. Furthermore, we found that the expression level of the *MED12* gene in the patient was significantly lower than the normal levels observed in her parents. The discrepancy in the levels of *MED12* was not related to age or sex under the comparison with a sex- and age-matched control group.

Since *MED12*-related syndromes exhibit X-linked inheritance, most female carriers are usually unaffected or with mild clinical symptoms (Graham and Schwartz, 2013; Prontera et al., 2016); however, the patient exhibited clinical features of *MED12*-related disease significantly. We predicted that the decrease in the expression of the *MED12* gene may be related to the X-chromosome inactivated by methylation. We subsequently conducted an X-chromosome inactivation study in the patient’s nuclear family, according to the method of methylation-sensitive PCR and analysis of androgen receptor CAG repeat polymorphism. The result showed that X-chromosome inactivation is skewed in this patent. We hypothesized that the causative pathogenesis in the patient may be related to the presence of the patient’s spontaneously mutated gene on the activated X-chromosome which was unable to produce sufficient levels of *MED12*, the critical component of the mediator complexes playing a key role in many signaling pathways, thus leading to the disease. This presumption should be further confirmed.

Based on previous studies, mutations of *MED12* may cause dysregulations in the SHH signaling pathway (Zhou et al., 2006; Srivastava et al., 2019), we examined the transcription levels corresponding to the three Gli3-dependent SHH signaling genes (Srivastava et al., 2019) in the nuclear family of the patient and a control group: *CREB5*, *BMP4*, and *NEUROG2*. The significant elevation in transcription levels of all these three genes in our patient with the *MED12* mutation may support the view that the mutation of *MED12* influences the regulation of the Gli3-dependent SHH signaling pathway to contribute to the craniofacial anomalies and multiple organ malformations of *MED12*-related XLID syndromes (Zhou et al., 2012; Srivastava et al., 2019).

To the best of our knowledge, the present study is the first novel splicing mutation reported in *MED12* gene-related disease in a female patient, with the clinical phenotype of *MED12*-related disorders. Our preliminary research put forward the possible mechanisms underlying the pathogenesis of our patient. The speculation of whether the patient’s spontaneously mutated *MED12* gene was on the activated X-chromosome remained to be further confirmed. The novel *de novo MED12* mutation in our female patient, which leads to significant clinical manifestations, raises the concern about the study in the mechanism of pathogenesis of heterozygous females. Furthermore, the induction of the patient’s naturally formed somatic cells into stem cells would be a valuable avenue for future research to explore possible functional changes induced by the mutation and further clarify the function of the *MED12* gene.

## Data Availability Statement

All datasets generated and analyzed for this study are included in the article/**Supplementary Material**.

## Author Contributions

CW and LL performed laboratory experiments and drafted the manuscript. YZ, FY, XNL, JX, and YW collected samples and analyzed clinical data. YC, YX, SW, XPL, and ZO analyzed the genomic data. LL and CW performed the formal analysis. LL, CW, and FY used the software and methodology. ZL, YZ, and XNL performed the validation. YC, KY, and YX edited the manuscript. Funds for the work were arranged by CMW and XS. All authors have approved the final manuscript.

## Funding

This work was supported by grants from the National Natural Science Foundation of China (No. 81650008), Shanghai Science and Technology Fund (No. 16410723400), Talent Introduction Fund of Shanghai Children’s Hospital (to YC), Key Subject Program from Shanghai Municipal Commission of Health and Family Planning (No. 2016ZB0102), Key disciplines of top priority in Shanghai (2017ZZ02019), and Shanghai Hospital Development Center (SHDC12015113) and Research project of Shanghai Children's Hospital (2016YMS005).

## Conflict of Interest

The authors declare that the research was conducted in the absence of any commercial or financial relationships that could be construed as a potential conflict of interest.

## Supplementary Material

Supplementary Figure 1 RT-qPCR was used to ascertain the expression levels of three target genes involved in the GLI3-dependent sonic hedgehog (SHH) signaling pathway: *CREB5*, *BMP4*, and *NEUROG2*. The expression levels of *CREB5*, *BMP4*, and *NEUROG* were all significantly enhanced in our patient relative to those in her parents and a sex- and age-matched control group.

Supplementary Figure 2
**(A)** Based on the previously reported variants, fifteen *MED12* mutations causing *MED12*-related disorders covering four phenotypes are clustered around the established functional Domains of *MED12*. (**B**) A conservative study on these 15 *MED12* missense mutation sites. Red arrows indicate heterozygous point mutation sites in humans relative to those in other species. These mutation sites were all located in a conservative position in evolution.

Supplementary Table 1 Primers used in this study are described in **Table 1**.

Supplementary Table 2 A review of previous studies on 15 meaningful missense mutations in *MED12* predicted to be pathogenic or likely pathogenic in the ClinVar Database (https://www.ncbi.nlm.nih.gov/clinvar) is summarized in **Table 2**.
