# Supplementary figures and images for: MED12-Related Disease in a Chinese Girl: Clinical Characteristics and Underlying Mechanism
Source: Front Genet. 2020 Feb 27;11:129. doi: 10.3389/fgene.2020.00129 (PMC7056888; doi:10.3389/fgene.2020.00129)

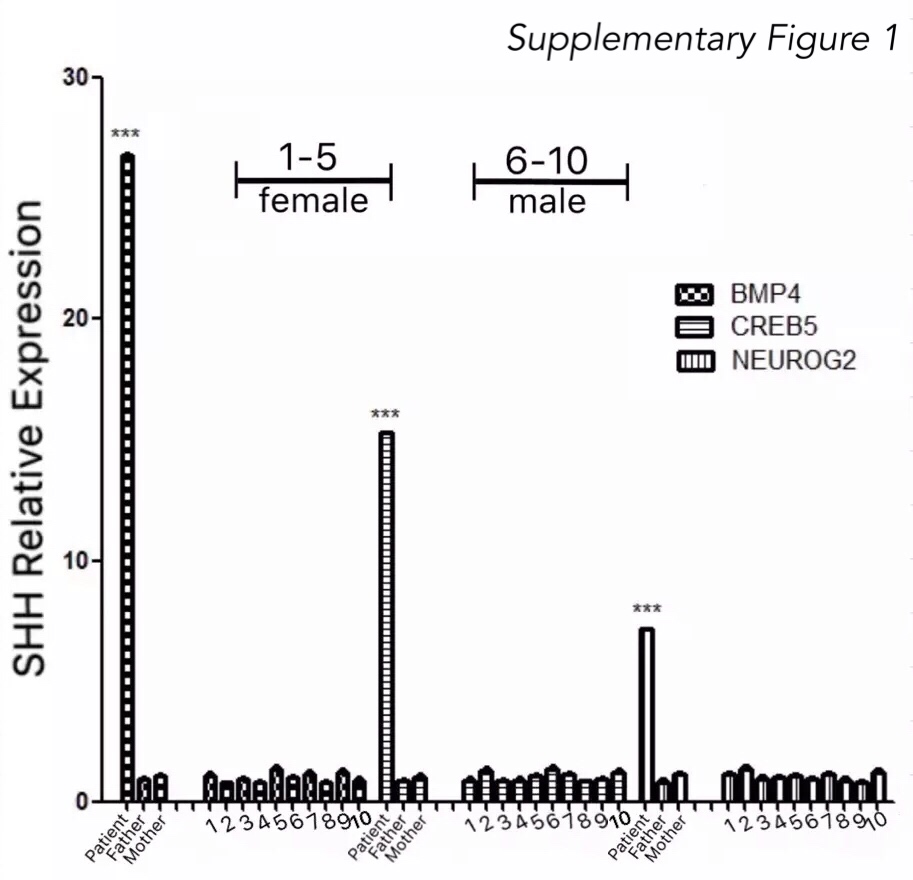

Supplement: Supplementary Figure 1 — RT-qPCR was used to ascertain the expression levels of three target genes involved in the GLI3-dependent sonic hedgehog (SHH) signaling pathway: CREB5, BMP4, and NEUROG2. The expression levels of CREB5, BMP4, and NEUROG were all significantly enhanced in our patient relative to those in her parents and a sex- and age-matched control group. [file Image_1.jpeg]

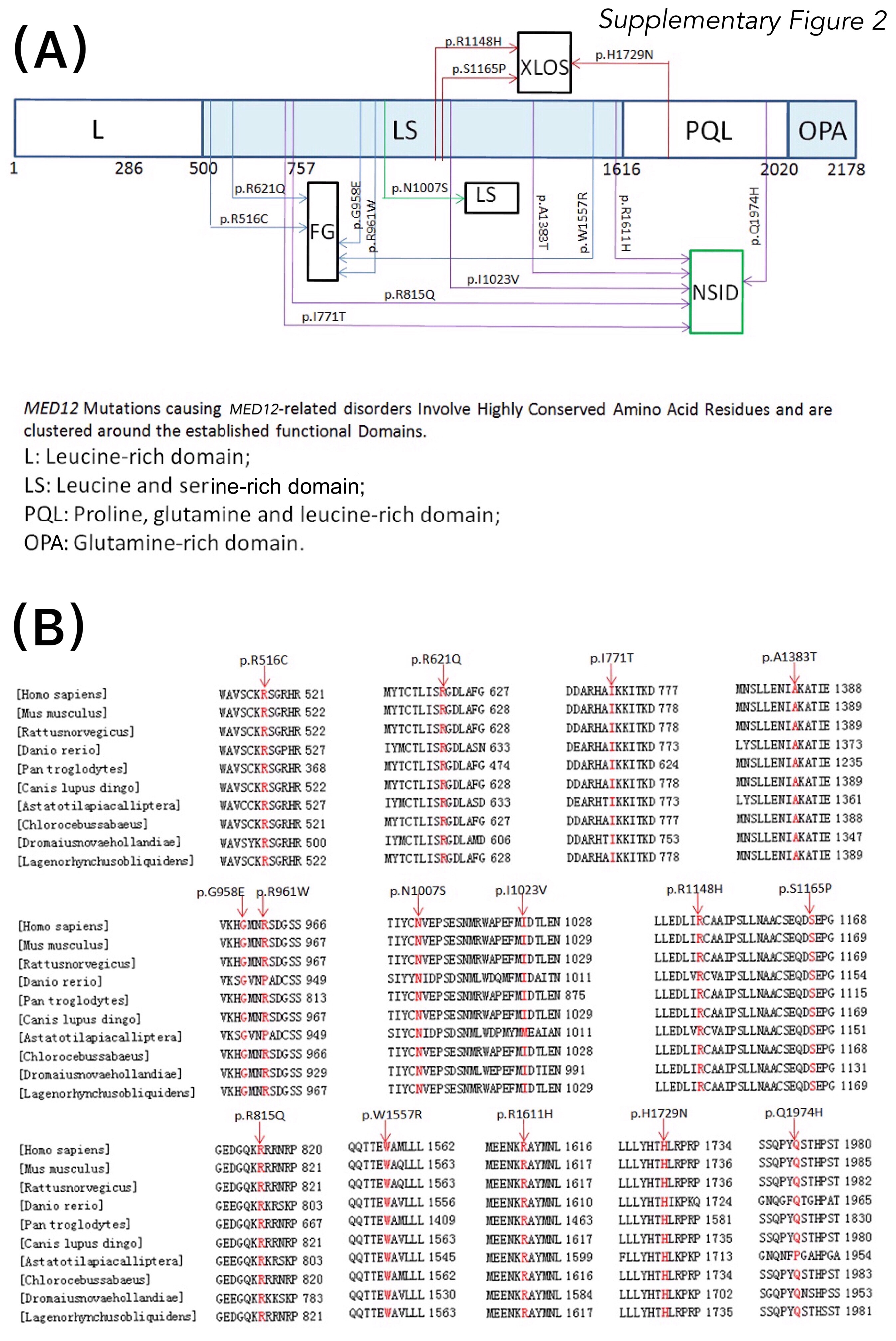

Supplement: Supplementary Figure 2 — (A) Based on the previously reported variants, fifteen MED12 mutations causing MED12-related disorders covering four phenotypes are clustered around the established functional Domains of MED12. (B) A conservative study on these 15 MED12 missense mutation sites. Red arrows indicate heterozygous point mutation sites in humans relative to those in other species. These mutation sites were all located in a conservative position in evolution. [file Image_2.jpg]
